# Temperature influences glyphosate efficacy on glyphosate-resistant and -susceptible goosegrass (*Eleusine indica*)

**DOI:** 10.3389/fpls.2023.1169726

**Published:** 2023-03-22

**Authors:** Wenlei Guo, Chun Zhang, Siwei Wang, Taijie Zhang, Xingshan Tian

**Affiliations:** ^1^ Guangdong Provincial Key Laboratory of High Technology for Plant Protection, Institute of Plant Protection, Guangdong Academy of Agricultural Sciences, Guangzhou, China; ^2^ Key Laboratory of Green Prevention and Control on Fruits and Vegetables in South China Ministry of Agriculture and Rural Affairs, Guangzhou, China

**Keywords:** low temperature, glyphosate, control efficacy, shikimate, absorption, translocation

## Abstract

Glyphosate has been widely used to control *Eleusine indica* and other weeds in South China for many years. Among the most troublesome weeds in South China, *E. indica* can remain alive all year round. However, the influence of temperature on glyphosate efficacy on *E. indica*, especially under days with fluctuating temperature, is unknown. This study evaluated the influence of two temperature regimes on glyphosate efficacy on glyphosate-resistant (R) and -susceptible (S) *E. indica* biotypes. Plants of the R and S biotypes were cultivated under two temperature regimes (high: 30°C/20°C day/night; low: 20°C/15°C day/night). Dose-response experiments showed improved efficacy of glyphosate at the low temperature compared with that at the high temperature for both biotypes. Based on the LD_50_ values, the R biotype was 8.9 times more resistant to glyphosate than the S biotype at the high temperature; however, the resistance index (R/S) decreased to 3.1 at the low temperature. At 4 days after glyphosate application, shikimic acid accumulation was greater at the low temperature than at the high temperature in plants of both biotypes, and the increase was higher in plants of the R biotype than in the S biotype. At a sublethal glyphosate dose (R: 400 g ai ha^−1^; S: 200 g ai ha^−1^), plants grown at the low temperature showed a strong decrease in leaf chlorophyll content and Fv/Fm value compared with those of plants grown at the high temperature and the untreated control. At 3 days after treatment, glyphosate absorption was similar between biotypes at the high temperature, but absorption decreased to 64.9% and 53.1% at the low temperature for the R and S biotypes, respectively. For both biotypes, glyphosate translocation from the leaf to the remainder of the plant was reduced at the low temperature compared with that at the high temperature. No differences in glyphosate translocation were observed between biotypes within each temperature regime. This is the first report on the effect of temperature on glyphosate efficacy on *E. indica*, and provides important insights for glyphosate application and resistance management.

## 1 Introduction

Since its commercialization in 1974, glyphosate has been widely used as a broad-spectrum, nonselective, post-emergence herbicide. It has been regarded as among the most important and successful herbicides (Duke and Powles, 2008). Glyphosate kills weeds by inhibiting the activity of 5-enolpyruvylshikimate-3-phosphate synthase (EPSPS) (Steinrucken and Amrhein, 1980), a key enzyme in the shikimate pathway in plants. If EPSPS is inhibited by glyphosate, one physiological response is rapid accumulation of shikimic acid (Holländer-Czytko and Amrhein, 1983), which is a biomarker for assessment of glyphosate efficacy in plants (Mueller et al., 2003; Zelaya et al., 2011). Elevated contents of shikimic acid in a treated plant indicates sensitivity to glyphosate, whereas a lack of or limited accumulation of shikimic acid indicates tolerance to glyphosate (Singh and Shaner, 1998; Burgos et al., 2013). Environmental factors may influence glyphosate efficacy and the level of glyphosate resistance in plants (Cerdeira et al., 2007). Among various environmental factors, temperature has received greater attention given that the absorption and translocation of glyphosate often varies considerably with temperature (Singh et al., 2020).

South China is located in the subtropical monsoon climate zone. Winter in South China is much warmer than in northern China. Taking Guangzhou as an example, from 1991 to 2020, the average maximum and average minimum temperatures were 20.7°C and 11.9°C in December, and 18.7°C and 10.6°C in January (http://www.tqyb.com.cn/gz/climaticprediction/static/). As a result, the life history of many weed species in South China differs from those in northern China. Goosegrass (*Eleusine indica*) is a troublesome grass weed that is widely distributed in most regions of China and other tropical or subtropical areas in the world (Chauhan and Johnson, 2008; Zhu et al., 2020). It often infests corn, vegetable, and fruit crops, and significantly reduces crop yield and quality (Zhang, 2003). In northern China, *E. indica* usually germinates from April to May and dies from October to November. However, because of the warm winter, *E. indica* can remain alive all year round and seeds can germinate in different months in South China. *E. indica* has shown 35%–60% germination at 15°C constant temperature or 15–20°C fluctuating temperature (Ismail et al., 2002; Chauhan and Johnson, 2008).

South China is one of the first areas in China to control weeds with glyphosate (Yang et al., 2012). Similar to other countries, the long-term application of glyphosate has resulted in development of glyphosate resistance in some weed species in China (Song et al., 2011; Chen et al., 2015; Zhang et al., 2015a; Deng et al., 2020; Li et al., 2022), especially in *E. indica*. Considering that paraquat use has been banned and the extent of glyphosate-resistant crops may increase significantly in the near future, it is likely that glyphosate will continue to play an important role in the nonselective herbicide market in China for the foreseeable future (Liu et al., 2021; Zhang et al., 2022). *E. indica* grows vigorously and shows strong competitive ability with crops in summer, so farmers in South China willingly spray glyphosate repeatedly in this period (Yang et al., 2012). Conversely, farmers usually do not apply glyphosate to control *E. indica* in low-temperature seasons because most *E. indica* plants are shorter and less competitive than other weeds or crops. Greater attention should be given to the vulnerable period of a weed’s lifecycle for effective weed control.

*E. indica* emerges at different times of the year in South China and the growth rate is relatively slow during the cold season, which may provide an excellent opportunity to control the plants by applying herbicide in winter or early spring. The plant biomass increases rapidly with the rise in temperature in spring and profuse seed production eventuates in the absence of control measures. To the best of our knowledge, the influence of temperature on the glyphosate efficacy or degree of glyphosate resistance in *E. indica* remains unknown. Therefore, the objectives of this study were to (1) determine the whole-plant dose-response of glyphosate-resistant and -susceptible biotypes of *E. indica* under high- and low-temperature regimes, (2) assess the changes in physiological indicators after glyphosate treatment under the different temperature regimes, including shikimic acid accumulation, chlorophyll content and chlorophyll fluorescence, and (3) compare absorption and translocation of glyphosate in the resistant and susceptible biotypes under the different temperature regimes.

## 2 Materials and methods

### 2.1 Plant material and temperature regimes

Two biotypes of *E. indica* were used in all experiments of this study. The glyphosate-resistant (R) biotype was purified from the R5 population described by Zhang et al. (2021). The R5 population was collected from a corn field in Baiyun district, Guangdong province (23°23′ N, 113°26′ E), where glyphosate had been applied consistently for at least 10 years. In the R5 population, plants surviving glyphosate treatment (1080 g ai ha^−1^) harbored different target-site mechanisms. The mother plants of the R biotype were related to *EPSPS* gene amplification, without *EPSPS* gene mutation (Zhang et al., 2021). Seeds of the susceptible (S) biotype were harvested from a nearby uncultivated area with the same geographic background as the R biotype (23°29′ N, 113°21′ E). The results of qPCR confirmed that the *EPSPS* gene copy number (relative to the acetolactate synthase gene) of the R biotype was 24 times higher than that of the S biotype (data not shown). The seeds were air-dried and stored in a cabinet at the ambient room temperature and 10%–14% relative humidity until use.

The seeds were rubbed with sandpapers to remove the thin pericarp, and then sown in plastic trays containing moistened nutrient soil. The trays were placed in a greenhouse with day/night temperature of 25°C/20°C, relative humidity of 70 ± 5%, and illuminated by natural sunlight. Seedlings of the R and S biotypes were transplanted individually into 200-mL pots containing nutrient soil. When most plants had attained the five-leaf stage, healthy plants of uniform size of each biotype were divided into two groups. The two groups were cultivated in different growth chambers under the same growing conditions, except that the day/night temperature regime was either 30°C/25°C (high temperature) or 20°C/15°C (low temperature). The plants were grown under a photoperiod of 12 h/12 h (day/night) with light intensity in the chambers of 200 μmol m^−2^ s^−1^ photon flux. The relative humidity in the growth chambers was maintained at 75 ± 5% throughout the experiment and the plants were watered every second day.

The plants were incubated in the respective growth chambers for 3 days to allow acclimation to the temperature regime before being treated with glyphosate.

### 2.2 Whole-plant dose-response experiment

After temperature acclimation for 3 days, the *E. indica* plants were treated with glyphosate (Roundup^®^, 41% isopropylamine salt of glyphosate; Bayer Crop Science, St. Louis, MO, USA) using a moving Teejet 9503EVS flat-fan nozzle cabinet sprayer (Beijing Research Center for Information Technology in Agriculture, Beijing, China) with a spray volume of 450 L ha^−1^ at 0.28 MPa. Glyphosate was applied at the rate of 0, 50, 100, 200, 400, 800, 1600, 3200, or 6400 g ai ha^−1^ for R biotype and 0, 12.5, 25, 50, 100, 200, 400, 800, or 1600 g ai ha^−1^ for the S biotype. Each treatment consisted three replications and each replication comprised five plants. At approximately 20 min after treatment, the plants were returned to the corresponding chambers. At 14 days after treatment (DAT), the number of surviving plant was recorded. Plants were classified as dead if they showed symptoms such as chlorosis and desiccation, and as alive if they were actively growing and tillering (Vila-Aiub et al., 2021). Two independent experiments were performed.

### 2.3 Shikimic acid accumulation

Glyphosate was applied to *E. indica* plants (grown under different temperature regimes) as described above at the rate of 0, 100, 200, 400, 800, 1600, 3200 g ai ha^−1^ for R biotype and 0, 25, 50, 100, 200, 400, 800 g ai ha^−1^ for S biotype.

The shikimate assay was conducted as described in Chen et al. (2015) with some modifications. Shikimic acid was extracted at 4 DAT. Tissue from fully expanded mature leaves (200 mg) was placed in a 2-mL centrifuge tube and then flash-cooled in liquid nitrogen before crushed using an automatic sample fast grinding machine (Shanghai Jingxin Industrial Development Co. Ltd, Shanghai, China). Immediately after crushing, 1 mL HCl (0.25 mol L^−1^) was added to the centrifuge tube. The tubes were vortexed for 10 s to ensure that the crushed tissue was fully dissolved in HCl. After centrifugation at 25,000 g for 20 min at 4 °C, 0.16 mL supernatant was transferred to a 5-mL centrifuge tube. Next, 1.6 mL periodic acid (1%, w/v) was added with sufficient mixing and the tube was incubated at room temperature. After incubation for 3 h, 1.6 mL NaOH (1 mol L^−1^) was added to the reaction mixture, followed by the addition of 0.96 mL glycine (0.1 mol L^−1^). The absorbance at 380 nm was measured using a Multiskan SkyHigh Microplate Spectrophotometer (Thermo Fisher Scientific (China) Co., Ltd, Shanghai, China). A standard curve was constructed after replacing the supernatant with a known concentration (6.25–800 μg mL^−1^) of shikimic acid solution.

### 2.4 Determination of chlorophyll content

As described in section 2.1, *E. indica* plants were grown under the high- and low- temperature regimes for 3 days before treatment with glyphosate. Commercial glyphosate at a sublethal dose was applied to plants of the R (400 g ai ha^−1^) and S (200 g ai ha^−1^) biotypes. Plants treated with deionized water were used as the untreated control. At 10 DAT, chlorophyll was extracted from 0.1 g leaf samples with 10 mL of a mixed solution of acetone and ethanol (1:1, v/v). The light absorbance at 647 nm and 663 nm was measured using a spectrophotometer as described in section 2.3. The chlorophyll content determinations were based on the methods and equations of Lichtenthaler (1987):


$$chlorophyll\;a\;\left(mg\;g^{-1}\right)\;=\;\left[\left(11.25\;\times A_{663}-\;2.79\;\times A_{647}\right)\;\times x\right]/\left(1000\;\times y\right)$$

$$chlorophyll\;b\;\left(mg\;g^{-1}\right)\;=\;\left[\left(21.5\;\times A_{647}-\;5.1\;\times A_{663}\right)\;\times x\right]/\left(1000\;\times y\right)$$

$$chlorophyll\;\left(a+b\right)\;\left(mg\;g^{-1}\right)\;=\;\left[\left(18.71\;\times A_{647}-\;6.15\;\times A_{663}\right)\;\times x\right]/\left(1000\;\times y\right)$$

where *A* is absorbance, *x* (mL) is the volume of the extraction solution, and *y* (g) is the fresh weight of the leaf sample.

### 2.5 Measurement of chlorophyll fluorescence

The glyphosate doses and treatments were identical to those of the chlorophyll content analysis. At 0, 2, 4, 6, and 8 DAT, the chlorophyll fluorescence was measured using a Dual-PAM-100 fluorescence measuring system (Heinz Walz GmbH, Effeltrich, Germany). At each time point, measurements were conducted at 09:00 to 12:00 after dark adaptation for 30 min. The investigation was performed with four replications corresponding to four individual plants. Data used for analysis were the leaf maximal quantum yields (Fv/Fm) of *E. indica* plants as described by Zhang et al. (2015b).

### 2.6 Absorption and translocation of glyphosate

Seeds of the R and S biotypes were germinated on 0.6% (w/v) agar-solidified Hoagland’s nutrient solution. Seedlings at two-leaf stage were transplanted into quadrate plastic containers (30 cm × 30 cm × 5 cm) containing Hoagland’s nutrient solution. Each seedling was clamped by a sponge and separated into a planting basket with a hole at the bottom. A daily supplement of the nutrient solution was guaranteed. The environmental conditions in this phase were identical to those described for the greenhouse.

When the plants attained the five-leaf stage, they were transferred to the growth chambers maintained at the aforementioned high- and low- temperature regimes. After 3 days, a commercial glyphosate solution was applied using a topical application method. Commercial glyphosate was diluted to 5000 mg L^−1^ (ai), then 8 μL of glyphosate solution was applied to the center of the fourth leaf of each plant using a micropipettor. The plants were returned to the growth chambers once the droplet had dried. Plants were harvested at 3 DAT. Each treatment comprised three replications each consisting of 25 plants. The treated leaf was excised with scissors. The treated leaves from one replication were rinsed in a glass test tube with 10 mL of wash solution (a mixture [1:1 v/v] of methanol and deionized water with 0.45% Tween-20) for 2 min to remove the unabsorbed glyphosate from the surface of the treated leaves (Ganie et al., 2017). The samples to be analyzed for each treatment were separated into treated leaves, other tissues (including shoots, roots and other leaves), and the wash solution.

For sample preparation, 0.5 mL of the wash solution was transferred to a fresh centrifuge tube, mixed with 0.5 mL methanol by vortexing for 1 min, and the mixed solution was filtered through a 0.22-µm 165 hydrophilic PTFE needle filter for subsequent liquid chromatography–tandem mass spectrometry (LC-MS/MS) analysis. The treated leaf and other parts of the plants were cut into pieces and weighed, then transferred to a 50-mL centrifuge tube, and 10 mL methanol was added. The mixture was homogenized for 3 min and centrifuged at 3200 g for 5 min. The supernatant was filtered through a 0.22-µm filter as described above and then subjected to LC-MS/MS analysis. The LC-MS/MS analysis of glyphosate and aminomethyl phosphonic acid (AMPA) was performed in accordance with the Chinese national standard SN/T 4655-2016 (General Administration of Quality Supervision, Inspection and Quarantine of the People’s Republic of China; https://www.sdtdata.com/)

### 2.7 Statistical analysis

Data for plant survival were transformed into a percentage of the untreated control before use to estimate the glyphosate dose required to cause 50% mortality (LD_50_) of the plants. The transformed data were then subjected to a four-parameter log-logistic regression model implemented in SigmaPlot 12.5 software: *y* = *c* + (*d − c*)/[1 + (*x*/LD_50_)*^b^*], where *y* is the plant survival rate, *c* is the lower limit around indefinitely high doses, *d* is the upper limit at indefinitely low doses, *b* is the slope around the LD_50_, and *x* is the glyphosate dose. The resistance index (RI) was calculated by dividing the LD_50_ value of the R biotype by that of the S biotype.

The means for shikimic acid accumulation, chlorophyll content, glyphosate absorption and translocation were compared, and the effects of temperature (or glyphosate dose) were analyzed by an unpaired Student’s test or one-way ANOVA (followed by a Fisher’s protected LSD test) using SPSS 21.0 software.

## 3 Results

### 3.1 Whole-plant dose-response experiment

**Figure 1:**
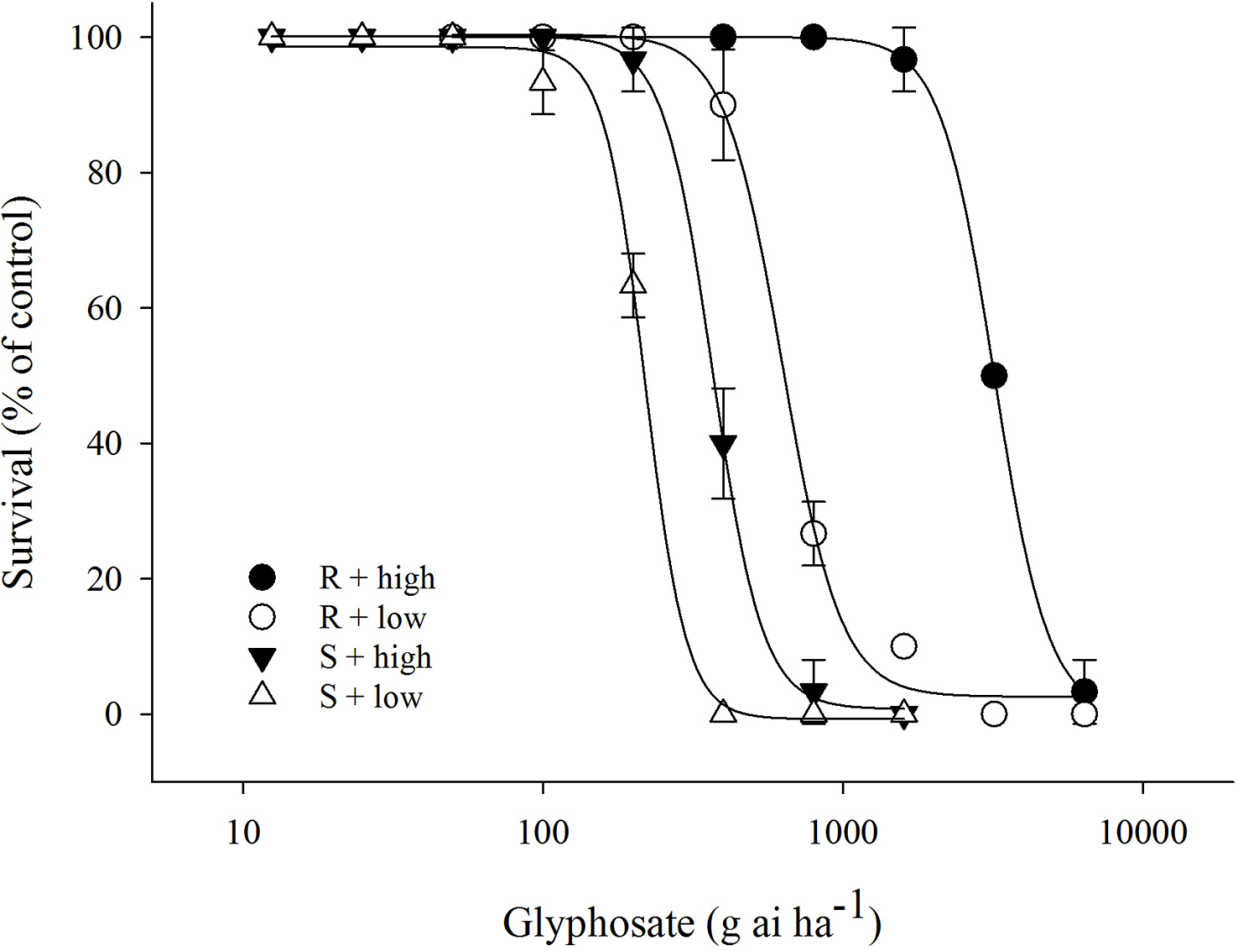
Plant survival response to glyphosate treatment in glyphosate-resistant (R) and -susceptible (S) *Eleusine indica* biotypes grown under high temperature (30°C/25°C day/night) and low temperature (20°C/15°C day/night) regimes. The experiment was repeated with similar results. Each data point is the mean ± standard error of three replicates from one experiment.

The efficacy of glyphosate on *E. indica* was distinctly influenced by temperature. Within a certain dose range, glyphosate killed more plants under the low-temperature regime than at the high-temperature regime (**Figure 1**). The LD_50_ values of the R biotype decreased from 3133.5 g ai ha^−1^ to 677.4 g ai ha^−1^ under the high- and low-temperature regimes, respectively (**Table 1**). The S biotype had LD_50_ values of 352.8 g ai ha^−1^ under the high-temperature regime and 219.2 g ai ha^−1^ under the low-temperature regime (**Table 1**). Therefore, the resistance index (R/S) dropped from 8.9 (high temperature) to 3.1 (low temperature) (**Table 1**).

**Table 1:** Estimated LD_50_ values in glyphosate-resistant (R) and -susceptible (S) *Eleusine indica* plants grown under high temperature (30°C/25°C day/night) and low temperature (20°C/15°C day/night) regimes.

Temperature regime	Biotype	LD_50_ (g ai ha^−1^)^a^	RI^b^
High	R	3133.5 ± 70.5	8.9
	S	352.8 ± 16.3	
Low	R	677.4 ± 20.6	3.1
	S	219.2 ± 2.8	

a LD_50_ is the glyphosate dose required to cause 50% mortality of the plants. Each value represents the mean ± standard error of two repeated experiments.

b RI (resistance index)=LD_50_ (R)/LD_50_ (S).

### 3.2 Shikimic acid accumulation

**Figure 2:**
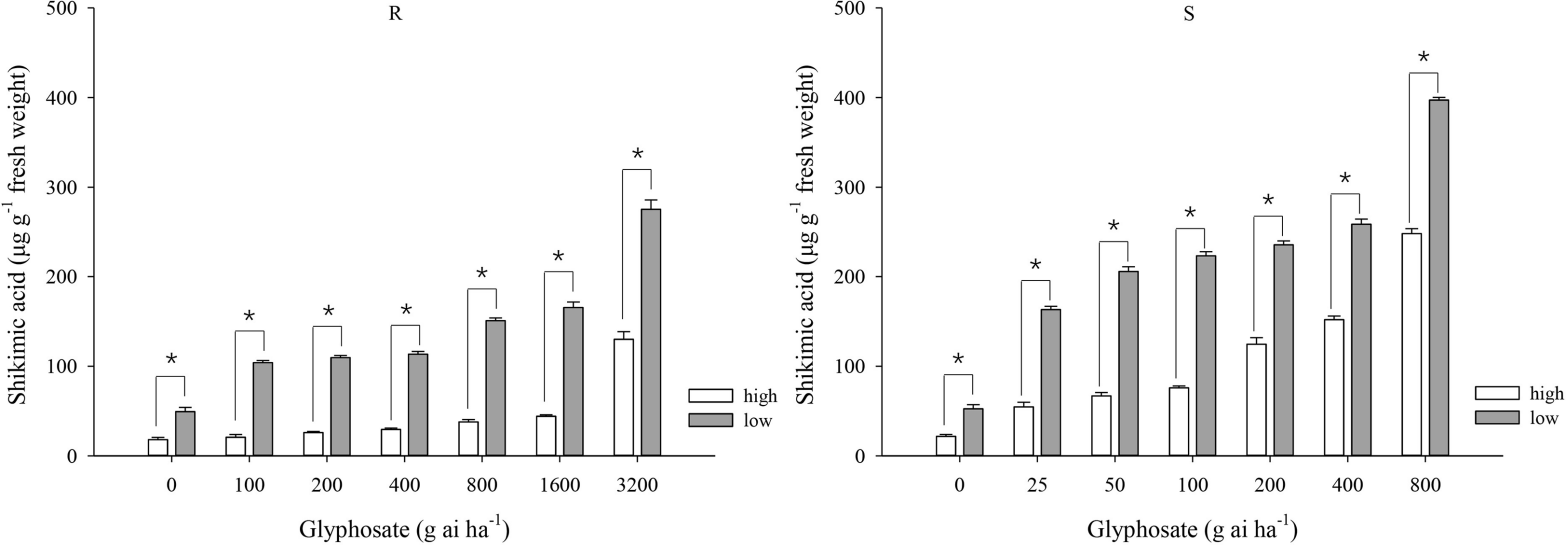
Shikimic acid accumulation in leaf tissue of glyphosate-resistant (R) and -susceptible (S) *Eleusine indica* biotypes at 4 days after glyphosate treatment under high temperature (30°C/25°C day/night) and low temperature (20°C/15°C day/night) regimes. Each data point is the mean ± standard error of three biological replications. An asterisk indicates a significant difference in shikimic acid content within the same glyphosate treatment between temperature regimes (Student’s *t*-test, α = 0.05).

At the same glyphosate dose, shikimic acid accumulation was greater under the low-temperature regime than under the high-temperature regime for both biotypes (**Figure 2**). At the high temperature, the amount of shikimic aicd in the R biotype showed muted increment from 18.0 to 44.1 μg g^−1^ at the glyphosate doses of 0–1600 g ai ha^−1^ and increased to 130 μg g^−1^ at 3200 g ai ha^−1^ (**Figure 2**), indicating high tolerance to glyphosate under this temperature regime. At the low temperature, the shikimic acid content gradually increased from 49.3 to 275.3 μg g^−1^ in the R biotype in response to glyphosate application (**Figure 2**). At glyphosate doses of 0–800 g ai ha^−1^, plants of the S biotype grown at the low temperature accumulated greater quantities of shikimic acid than those grown at high temperature (52.4–397.2 and 21.7–248.2 μg shikimic acid g^−1^ fresh weight, respectively) (**Figure 2**).

Irrespective of the temperature regime, the S biotype always accumulated greater shikimic acid contents than plants of the R biotype, although the difference between the two biotypes was narrower at the low temperature. For example, S plants accumulated 151.9 μg shikimic acid g^−1^ fresh weight at the high temperature under the glyphosate dose of 400 g ai ha^−1^ (**Figure 2**), which was 4.1 times higher than that for the R plants; however, although S plants accumulated substantially more shikimic acid (258.6 μg g^−1^) at the low temperature under the same glyphosate treatment (**Figure 2**), the amount was only 1.3 times higher than that accumulated by the R plants.

### 3.3 Chlorophyll content

In the 0 g ai ha^−1^ glyphosate treatment, temperature regime did not influence the leaf chlorophyll contents of *E. indica* plants of either the R or S biotype (**Table 2**). In addition, the chlorophyll a and chlorophyll b contents increased in response to a sublethal dose of glyphosate (R: 400 g ai ha^−1^; S: 200 g ai ha^−1^) under the high-temperature regime compared with the untreated control (**Table 2**). However, the leaf chlorophyll contents of the R and S biotypes under the low-temperature regime decreased dramatically under the same glyphosate treatment (**Table 2**).

**Table 2:** Influence of glyphosate on leaf chlorophyll content of glyphosate-resistant (R) and -susceptible (S) *Eleusine indica* plants grown under high temperature (30°C/25°C day/night) and low temperature (20°C/15°C day/night) regimes.

Biotype	Glyphosate dose(g ai ha^−1^)	Temperature regime	Chlorophyll a (mg g^−1^)^a^	Chlorophyll b (mg g^−1^)^a^	Chlorophyll a+b (mg g^−1^)^a^
R	0	High	1.00 ± 0.03b	0.32 ± 0.02ab	1.32 ± 0.05b
		Low	0.96 ± 0.02b	0.29 ± 0.02b	1.26 ± 0.05b
	400	High	1.08 ± 0.01a	0.35 ± 0.01a	1.43 ± 0.02a
		Low	0.79 ± 0.02c	0.25 ± 0.02c	1.04 ± 0.04c
S	0	High	0.93 ± 0.02b	0.29 ± 0.02b	1.22 ± 0.03b
		Low	0.90 ± 0.01b	0.26 ± 0.03b	1.16 ± 0.04b
	200	High	1.02 ± 0.03a	0.38 ± 0.02a	1.40 ± 0.05a
		Low	0.48 ± 0.05c	0.16 ± 0.01c	0.65 ± 0.07c

a Chlorophyll extraction was conducted at 10 days after glyphosate treatment. Within the same biotype, values in the same column followed by different lowercase letter are statistically different (Fisher’s protected LSD, α = 0.05). Each value represents the mean ± standard error.

### 3.4 Chlorophyll fluorescence

**Figure 3:**
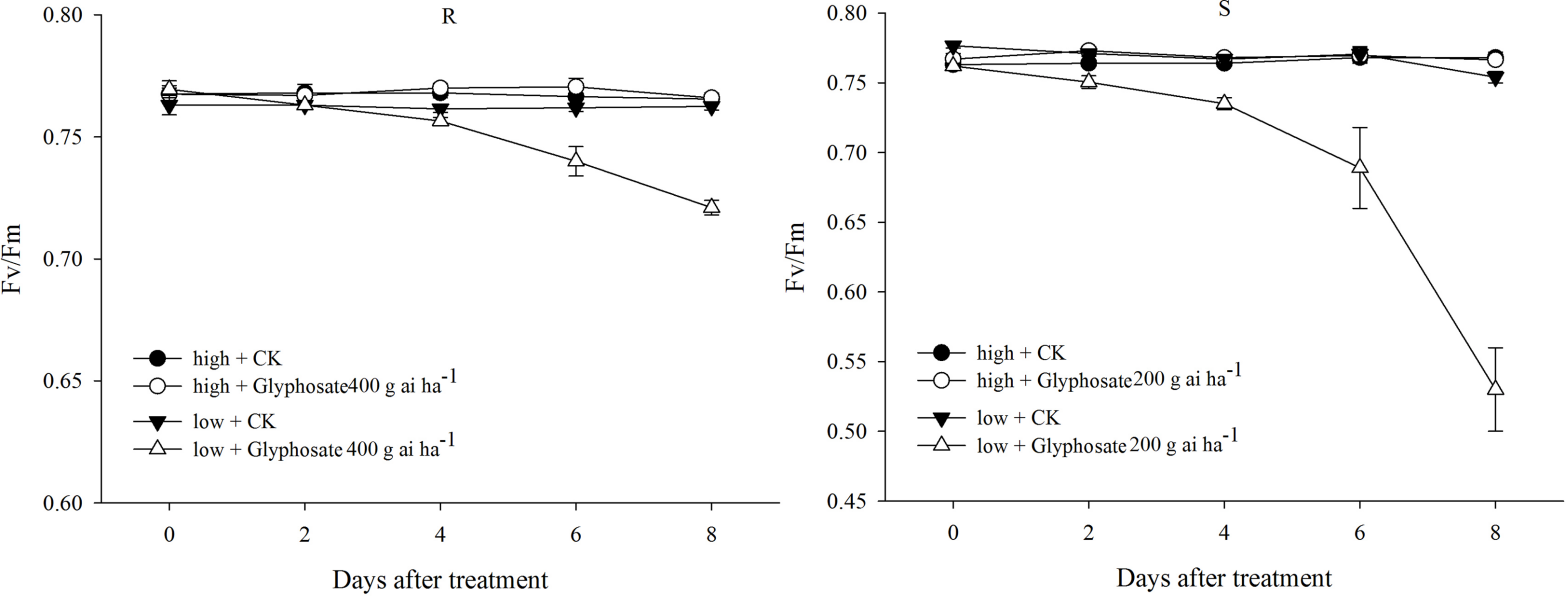
Fv/Fm value of glyphosate-resistant (R) and -susceptible (S) *Eleusine indica* biotypes after glyphosate treatment under high temperature (30°C/25°C day/night) and low temperature (20°C/15°C day/night) regimes. Each data point is the mean ± standard error of four biological replications.

In the control, only slight changes in Fv/Fm of the R and S biotypes were detected under the two temperature regimes (**Figure 3**). A sublethal dose of glyphosate (R: 400 g ai ha^−1^; S: 200 g ai ha^−1^) did not cause a distinct decline in Fv/Fm in the R and S biotypes grown at the high temperature (**Figure 3**). However, different responses were observed under the low-temperature regime; the Fv/Fm values of the R and S biotypes dramatically decreased to 0.72 and 0.53 at 8 DAT with the sublethal dose of glyphosate (**Figure 3**), which were 93% and 69% of the initial values, respectively.

### 3.5 Absorption and translocation of glyphosate

**Figure 4:**
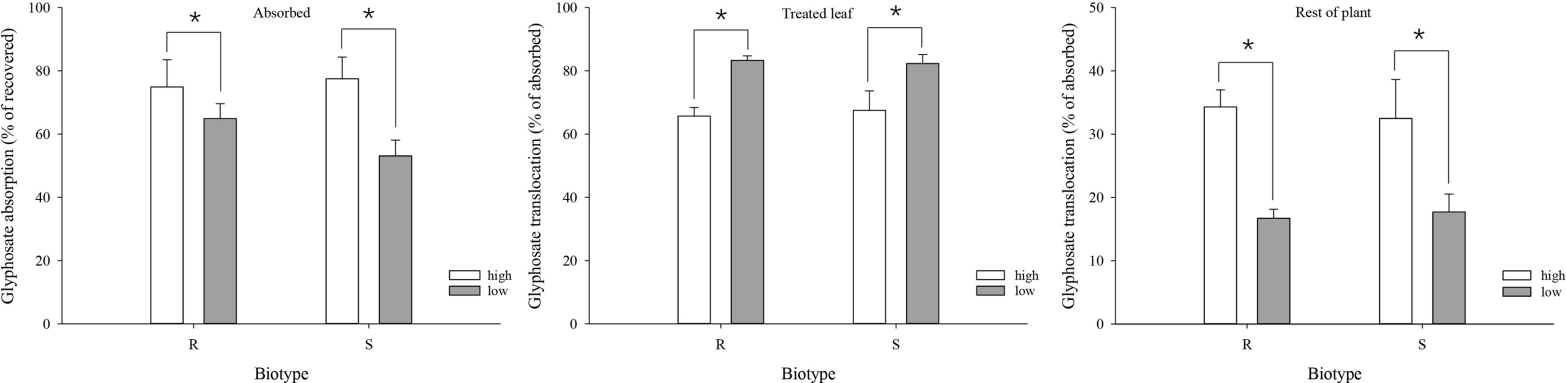
Glyphosate absorption and translocation in glyphosate-resistant (R) and -susceptible (S) *Eleusine indica* biotypes at 3 days after glypphosate treatment under high temperature (30°C/25°C day/night) and low temperature (20°C/15°C day/night) regimes. Each data point is the mean ± standard error of three biological replications. An asterisk indicates a significant difference between temperature regimes (Student’s *t*-test, α = 0.05).

At 3 DAT, the mean absorption of glyphosate was 74.9% and 77.5% under the high-temperature regime, and decreased to 64.9% and 53.1% under the low-temperature regime, in the R and S biotypes, respectively (**Figure 4**). As a result, absorption was significantly higher under the high-temperature regime than under the low-temperature regime for both biotypes (**Figure 4**).

Significant differences in glyphosate translocation were detected between the temperature regimes, but not between the biotypes within the same temperature regime. Both biotypes translocated a smaller proportion of the absorbed glyphosate from the treated leaf to the rest of the plant under the low-temperature regime. At 3 DAT, 65.7% (R) and 67.5% (S) of the absorbed glyphosate were retained in the treated leaf under the high-temperature regime compared with 83.3% (R) and 82.3% (S) under the low-temperature regime (**Figure 4**). The amount of glyphosate detected in the rest of the plant was 34.3% (R) and 32.5% (S) under the high-temperature regime compared with 16.7% (R) and 17.7% (S) at the low temperature (**Figure 4**)

In the experiment, AMPA was not detected in the treated leaf or in the remainder of the plant, and only a small amount of AMPA were detected in the wash solution (data not shown).

## 4 Discussion

The efficacy of glyphosate at different temperatures varies depending on the weed species and/or populations. The present study demonstrated that both the R and S biotypes of *E. indica* were more sensitive to glyphosate when grown at a low temperature. Compared with the S biotype, the sensitivity to glyphosate of the R biotype was more strongly elevated at the low temperature, resulting in reduced resistance level to glyphosate. Populations of *Echinochloa colona*, a graminaceous weed with a growing season similar to that of *E. indica*, displayed different trends in glyphosate tolerance at 20°C and 30°C. The efficacy of glyphosate on the sensitive *E. colona* population was not affected by temperature, but increased efficacies were observed at low temperature on six glyphosate-resistant *E. colona* populations (Nguyen et al., 2016). Tanpipat et al. (1997) demonstrated that increase in temperature reduces the control effect of glyphosate on *E. colona* under field conditions. An additional grass weed, *Lolium rigidum*, is less tolerant to glyphosate at a low temperature (Powles et al., 1998; Vila-Aiub et al., 2013). In addition, three glyphosate-resistant *Conyza* species (*C. sumatrensis*, *C. bonariensis*, and *C. canadensis*) show a reduction in glyphosate resistance at a low temperature (Ge et al., 2011; Okumu et al., 2019; Palma-Bautista et al., 2019). It is interesting that the efficacy of glyphosate on a sensitive biotype of *C. sumatrensis* decreases, but that on a resistant biotype increases, at a low temperature (Palma-Bautista et al., 2019). However, giant ragweed (*Ambrosia artemisiifolia*) and common ragweed (*Ambrosia trifida*) show higher mortality and biomass reduction at a high temperature regardless of the degree of susceptibility or resistance to glyphosate (Ganie et al., 2017).

The background shikimic acid amounts of the R and S biotypes of *E. indica* were similar in untreated plants under the same temperature regime, which increased from 18.0–21.7 μg g^−1^ at the high temperature to 49.3–52.4 μg g^−1^ at the low temperature. According to Mueller et al. (2008, 2011), shikimic acid concentrations are less than 80 μg g^−1^ in untreated plants of *E. indica*, and are usually less than 100 μg g^−1^ in nine other weedy species. Hence, it is reasonable to assume that the low-temperature treatment enhanced the background amount of shikimic acid in untreated *E. indica* plants, which was nonfatal to the plants. With exposure to glyphosate, shikimic acid may accumulate rapidly in the plant if EPSPS activity is partially or entirely inhibited by the herbicide. In most studies that have performed shikimate assays, glyphosate was applied at the field rate and the shikimic acid amount was measured during the period of 0–8 DAT. Generally, the shikimic acid amount is probably close to the maximum at 4–6 DAT for most species, including *E. indica* (Mueller et al., 2008; Mueller et al., 2011). In the present study, *E. indica* plants were incubated for 4 days at the different temperatures to evaluate the effect of temperature on shikimic acid accumulation after glyphosate treatment. The data for shikimic acid accumulation were in accordance with the results of the whole-plant experiment, in that a lower dose of glyphosate at a low temperature could achieve a comparable effect to that with a higher dose at a high temperature. In addition, the increasing difference in shikimic acid accumulation between the high- and low-temperature regimes was higher in the R biotype than in the S biotype of *E. indica*. The resistant biotype of *C. bonariensis* accumulated approximately five times more shikimic acid at 15 °C than at 27 °C, whereas the susceptible biotype only doubled the amount of shikimic acid (Okumu et al., 2019). In *C. sumatrensis*, the differences in shikimic acid accumulation between resistant and susceptible biotype were more divergent when plants were grown at a high temperature (Palma-Bautista et al., 2019). In *E. colona*, however, less shikimic acid was accumulated at a low temperature (20 °C) than at a high temperature (30 °C) (Nguyen et al., 2016).

The influences of glyphosate on chlorophyll content reflects the doses applied. Previous studies confirm that a lethal dose of glyphosate sharply reduces the chlorophyll content of plants (Chen et al., 2015). In contrast, plants treated with sublethal doses of glyphosate may have a higher chlorophyll content than the untreated control. For example, the chlorophyll content of *Chenopodium album* plants treated with a glyphosate dose of 90 and 180 g ai ha^−1^ was significantly higher than that of the untreated control (Ketel, 1996). Similar results were observed in *Amaranthus retroflexus* (Meseldžija et al., 2020). It is noteworthy that the stimulatory effect of glyphosate may differ with environmental factors (Belz and Duke, 2014). In the present study, when grown under the high temperature regime, the *E. indica* plants exposed to a sublethal dose of glyphosate had a higher leaf chlorophyll content than that of the untreated plants, but the stimulatory effect was not observed for plants grown under the low temperature regime, indicating that certain physiological processes may be impaired. The Fv/Fm value can be used as an indicator of tolerance to a herbicide and other environmental stress. A previous study of our team showed that a sublethal dose of glyphosate does not result in an obvious reduction in Fv/Fm in *E. indica* plants (Zhang et al., 2015b), which is consistent with the results obtained under the high-temperature regime in the present study. However, a sublethal dose caused a significant decrease in Fv/Fm in plants grown under the low temperature regime, especially for the S biotype, indicating that the functioning of photosystem II may be severely damaged. The observation that *E. indica* plants treated with sublethal doses of glyphosate showed reductions in chlorophyll content and Fv/Fm at a low temperature may be further evidence for the influence of temperature on glyphosate efficacy.

In many plant species, temperature has been reported to influence the absorption and/or translocation of glyphosate. In *E. colona*, glyphosate absorption was reduced with increase in temperature, whereas translocation from the treated leaf was not affected by temperature (Nguyen et al., 2016). The indication that glyphosate absorption and translocation are enhanced at a low temperature has been observed in many other plant species, such as *C. sumatrensis* (Palma-Bautista et al., 2019), Glycine max (McWhorter et al., 1980), and *Kochia scoparia* (Ou et al., 2018). In contrast, McWhorter et al. (1980) reported that *Sorghum halepense* absorbed two times the amount of glyphosate at 30 °C compared with at 24°C, and the degree of glyphosate translocation was also increased. Similarly, in *Brunnichia ovata*, Reddy (2000) noted that a greater amount of glyphosate was absorbed and translocated under a higher temperature. In the present study, it is interesting that increase in the glyphosate efficacy on *E. indica* was accompanied by reduced glyphosate absorption and translocation at a low temperature. A reduction in glyphosate absorption and translocation has been reported to confer glyphosate resistance in some weed species (Sammons and Gaines, 2014; Gaines et al., 2020). Hence, the increase in glyphosate efficacy on *E. indica* at a low temperature was likely caused by other mechanisms. A low temperature is not optimal for growth of *E. indica*; therefore, we hypothesize that an extended low-temperature treatment may disrupt certain physiological processes, resulting in reduced glyphosate tolerance. For example, if *EPSPS* gene expression of *E. indica* is suppressed under a low temperature, the amount of EPSPS enzyme would be reduced in cells and a lower glyphosate concentration would be required to inhibit its activity.

The present findings revealed that temperature impacted on the efficacy of glyphosate for control of the troublesome weed *E. indica* in South China. Therefore, temperature should be considered before glyphosate application, especially for applications in winter or early spring in South China, where strong fluctuations in the spring temperature usually occur. For control of *E. indica*, the temperature forecast in the days subsequent to glyphosate application should be colder for improved efficacy. The present research was conducted under artificial controlled conditions with precise temperatures and relative humidity; thus, the results may vary under field conditions owing to the complex variability of environmental factors, including temperature, relative humidity, wind, and light (Ramsey et al., 2005; Varanasi et al., 2016). In addition, considering that a premix formulation or tank mixture of glyphosate and other herbicides (such as glufosinate, dicamba, oxyfluorfen and flumioxazin) is increasingly popular (Beckie, 2011; Liu et al., 2021), the effect of temperature on the efficacy of other herbicides should also be considered. Further studies are needed to evaluate the combined effect of temperature and other environmental factors on weed control efficacy under field conditions. Moreover, molecular studies, such as analysis of gene expression for target enzymes or metabolic enzymes under different environmental conditions, may provide additional evidence to explain the variable response to herbicide application under different environmental conditions.

## Data availability statement

The raw data supporting the conclusions of this article will be made available by the authors, without undue reservation.

## Author contributions

WG and XT conceived and designed the study. WG, SW, and TZ performed experiments, acquired data and completed formal analysis. WG prepared the original draft. CZ and XT reviewed the manuscript, supervised and acquired the funding. All authors contributed to the article and approved the submitted version.
